# A Numerical Study of Elastic Wave Arrival Behavior in a Naturally Fractured Rock Based on a Combined Displacement Discontinuity-Discrete Fracture Network Model

**DOI:** 10.1007/s00603-022-03180-4

**Published:** 2022-12-19

**Authors:** Shuaifeng Wang, Zixin Zhang, Xin Huang, Qinghua Lei

**Affiliations:** 1 Department of Geotechnical Engineering, College of Civil Engineering, Tongji University, Shanghai, People’s Republic of China; 2 Key Laboratory of Geotechnical and Underground Engineering, Ministry of Education, Tongji University, Shanghai, People’s Republic of China; 3 Department of Earth Sciences, ETH Zürich, Zurich, Switzerland; 4 Department of Earth Sciences, Uppsala University, Uppsala, Sweden

**Keywords:** Wave transport, Arrival behavior, Natural fracture network, Fractal, Self-affine

## Abstract

The arrival behavior of elastic waves in a naturally fractured rock is studied based on numerical simulations. We use the discrete fracture network method to represent the distribution of a natural fracture system and employ the displacement discontinuity method to compute the propagation of elastic waves across individual fractures. We analyze macroscopic wavefield arrival properties collectively arising from the interaction between elastic waves and numerous fractures in the system. We show that the dimensionless angular frequency *ῶ* = *ωZ*/*κ* exerts a fundamental control on the arrival behavior of a plane wave traveling through the fractured rock, where *ω*, *Z*, and *κ* are the angular frequency, seismic impedance, and fracture stiffness, respectively. An asynchronous arrival phenomenon of the wave energy occurs and becomes more significant with an increased *ῶ*. Two regimes are identified according to the two-branch dependency of the fractal dimension *D* of the FFAW on *ῶ*, where the wave arrival behavior is within a non-fractal regime for *ῶ* smaller than the critical frequency *ῶ*_c_ ≈ 1.0, and enters the fractal regime for *ῶ* ≥ *ῶ*_c_. The self-affine properties of the FFAW, i.e., the roughness exponent *α* and the correlation length *l*_c_, both linearly decrease as a function of the exponent *ξ* (with *ῶ* = 10^*ξ*^) in the fractal regime. Early breakthrough of wave transport occurs in regions with relatively low fracture density, while late-time arrival happens in regions of high fracture density.

## Highlights

- Elastic wave transport in a naturally fractured rock is studied based on numerical simulations.
- Asynchronous arrival phenomenon of the wave energy becomes more significant with an increased dimensionless angular frequency *ῶ*.
- The front of first arrival waves is characterized by properties correlated with *ῶ* in the fractal regime.
- Early breakthrough of wave energy occurs in regions of low fracture density, while late-time arrival happens in regions of high fracture density.

## Introduction

Rock masses often contain numerous fractures which exist over a wide range of length scales from micrometers to kilometers (Sornette and Davy 1991; Ouillon et al. 1996; Bonnet et al. 2001; Bour et al. 2002; Davy et al. 2010; Lei et al. 2017). These natural fractures, with complicated geometrical and topological patterns, may significantly affect the seismic wave transport in the subsurface leading to scattering and attenuation (Aki 1980; Adler and Thovert 1999; Sato and Fehler 2009). This problem has attracted great attention from different disciplines, such as geophysics, seismology, rock mechanics, and earthquake engineering (Adler and Thovert 1999; Toomey et al. 2002; Spanos 2009; Sahimi 2011; Khoshhali and Hamzehpour 2015; Fan et al. 2018; Feng et al. 2020; Zhang et al. 2021). In addition, natural fractures often dominate the thermo-hydro-mechanical behavior of geological formations, which are highly relevant to many rock engineering applications such as underground excavation, hydrocarbon recovery, and nuclear waste disposal (Tsang 1999). However, it is very difficult to directly characterize the geometrical and mechanical properties of natural fractures due to their deeply buried nature (Lei et al. 2017). Studying wave propagation in fractured rocks is considered as a possible solution to this issue by imaging subsurface fractures using elastic waves (Bleistein et al. 2001; Allaei and Sahimi 2006) that are highly sensitive to the geometrical distribution and mechanical properties of fractures in rock (Pyrak-Nolte et al. 1990; Pyrak-Nolte 1996; Zhao and Cai 2001; Rubino et al. 2013; Shi and Lei 2018; Lei and Sornette 2021a). Thus, understanding how waves evolve in fractured rocks and quantifying the relationship between wavefield characteristics and fracture network properties are fundamentally important.

Over the past decades, extensive research has been done to investigate wave propagation in fractured rocks based on theoretical analysis (Schoenberg 1980; Hudson 1981; Crampin 1984; Pyrak-Nolte and Cook 1987; Shapiro and Kneib 1993; Pyrak-Nolte and Nolte 1995; Zhao and Cai 2001; Perino et al. 2010; Li 2013; Li et al. 2014, 2015; Fan et al. 2018, 2021), laboratory experiments (Pyrak-Nolte et al. 1990; Pyrak-Nolte 1996; Huang et al. 2014; Chen et al. 2015, 2016; Zhu et al. 2015; Liu et al. 2017; Li et al. 2017, 2019; Modiriasari et al. 2020), and numerical simulations (Vlastos et al. 2003, 2007; Wang et al. 2006, 2022; Deng et al. 2012; Fan et al. 2013; Fu et al. 2015; Yousef and Angus 2016; Chen et al. 2019; Zhu et al. 2020; Lei and Sornette 2021a, b; Yang et al. 2021; Sawayama et al. 2022). The elementary scenario for studying wave propagation in fractured rock is the transmission of wave energy across a single fracture, which is controlled by the fracture stiffness, wave frequency, and matrix properties (Schoenberg 1980; Pyrak-Nolte et al. 1990). For an infinite fracture with a negligible aperture, the displacement discontinuity method was commonly used to study the reflection, transmission, and refraction of elastic waves passing through a fracture at a normal or oblique direction (Schoenberg 1980; Pyrak-Nolte et al. 1990; Gu et al. 1996; Li and Ma 2010). When the fracture aperture is non-negligible with respect to the wavelength and there are infilling materials inside the fracture, a continuum representation of the fracture of a finite thickness connecting the adjacent rock walls may need to be used (Li et al. 2014). In addition, the method of characteristics was also used to investigate elastic waves across an infinite fracture based on one-dimensional wave propagation assumption (Bedford and Drumheller 1994; Cai and Zhao 2000; Fan et al. 2018). If waves encounter a finite-sized fracture, wave scattering and diffraction may occur at fracture tips (Liu and Zhang 2001; Rodriguez-Castellanos et al. 2006; Zhu et al. 2020; Lei and Sornette 2021a, 2021b). Many previous studies further explored the wave behavior in simplified fracture networks either with a constant length distribution (Kelner et al. 1999a; Deng et al. 2012; Hamzehpour et al. 2014, 2016; Khoshhali and Hamzehpour 2015; Chai et al. 2016) or a parallel/orthogonal directional configuration (Kelner et al. 1999b; Vlastos et al. 2003; Huang et al. 2014; Chen et al. 2015; Shao and Pyrak-Nolte 2016; Yousef and Angus 2016).

However, fracture systems in nature are much more complex than those idealized scenarios, because they usually contain a network of intersecting fractures of variable lengths, locations, and orientations (Odling 1997; Adler and Thovert 1999; Lei and Wang 2016; Lei et al. 2017; Afshari Moein et al. 2019). Elastic waves in natural fracture systems involving systematic fracture sets and variable fracture lengths may exhibit much more complicated wavefield phenomena compared to those in idealized fracture networks. A typical example is the velocity anisotropy in the crust (Liu and Crampin 1990; Crampin and Lovell 1991; Liu et al. 2004; Fontaine et al. 2009; Al-Harrasi et al. 2011; Frietsch et al. 2015; Robinson et al. 2020) intrinsically caused by the complicated spatial distribution of fractures (Kohler et al. 2003; Matonti et al. 2017; Zhang et al. 2020), which often results in a highly variable arrival behaviors of seismic waves (Majer et al. 1988; Spetzler and Snieder 2001; Santos et al. 2015; Shao and Pyrak-Nolte 2016; Xu et al. 2018; Najdahmadi et al. 2018). Studying the linkage between the wave arrival behavior and the fracture network distribution can be useful for characterizing crustal heterogeneities in many rock engineering applications.

In this paper, a series of 2D numerical simulations of plane wave propagation through a naturally fractured rock is conducted to explore the elastic wave transport and arrival behavior. We focus on the elastic scattering effect by fractures in rock under dry conditions, while the absorption effect related to inelastic processes is omitted. The rest of the paper is organized as follows. Section 2 describes the mathematical formulation, model setup, and analysis procedure. Section 3 shows the simulation results of the spatiotemporal wavefield evolution and the characterization results of wave arrival properties. We discuss the potential implications of our findings in Sect. 4 and present main conclusions in Sect. 5.

## Methodology

### Mathematical Formulation

The equation of dynamic equilibrium that governs elastic wave propagation in solids is given as:

1 $${\mathbf{M}}\frac{{\partial^{2} {\mathbf{u}}}}{{\partial t^{2} }} + {\mathbf{Ku}} = {\mathbf{F}},$$

where **M** and **K** are the mass and stiffness matrices, respectively, **u** is the displacement, *t* is the time, and **F** is the external force. In this paper, the 2D time-domain elastic wave equation is solved using the finite element method (COMSOL 2019).

To simulate wave propagation in a 2D unbounded medium, the stiffness reduction method (Pettit et al. 2014) is implemented to suppress unwanted wave reflections at the boundaries of the finite-sized study area. An absorbing layer with a thickness of 1.5*λ* is placed at the artificial boundaries, where *λ* is the wavelength of the incident wave. The governing equation for waves in the absorbing layer is given as:

2 $${\mathbf{M}}\frac{{\partial^{2} {\mathbf{u}}}}{{\partial t^{2} }} + {\mathbf{C}}^{*} \frac{{\partial {\mathbf{u}}}}{\partial t} + {\mathbf{K}}^{*} {\mathbf{u}} = {\mathbf{F}},$$

where C^*^ = *η***M** and K^*^ = *ς***K** are the damping and stiffness matrices, respectively, with *η* and *ς* being the mass proportional damping coefficient and the stiffness reduction coefficient, respectively. Within the absorbing layer, the two coefficients are assigned as *η*(*χ*) = *ω*(*χ*/(1.5*λ*))^3^ and *ς*(*χ*) = exp(–*ψ*(*χ*)*kχ*), where *χ* is the distance away from the boundary of the study area, *ω* is the angular frequency of the incident wave, *k* is the incident wavenumber, *ψ*(*χ*) = *ψ*_max_(*χ*/(1.5*λ*))^3^ is the attenuation factor, and *ψ*_max_ = –ln(*ε*/(1.5*λk*)) is the maximum attenuation factor with *ε* taking a small value, e.g., 0.01.

The elastic wave propagation across natural fractures is modeled based on the displacement discontinuity method (Schoenberg 1980), where each fracture is represented as an internal interface assuming a vanishing thickness and a non-welded contact. Across the fracture, the stress is continuous, while the displacement is discontinuous (Pyrak-Nolte et al. 1990), such that:

3 $$\left\{ {\begin{array}{*{20}l} {\sigma_{{\text{n}}}^{ + } = \sigma_{{\text{n}}}^{ - } } \hfill \\ {\sigma_{{\text{s}}}^{ + } = \sigma_{{\text{s}}}^{ - } } \hfill \\ {u_{{\text{n}}}^{ + } - u_{{\text{n}}}^{ - } = \sigma_{{\text{n}}} /\kappa_{{\text{n}}} } \hfill \\ {u_{{\text{s}}}^{ + } - u_{{\text{s}}}^{ - } = \sigma_{{\text{s}}} /\kappa_{{\text{s}}} } \hfill \\ \end{array} } \right.,$$

where *σ*, *u*, and *κ* are the stress, displacement, and specific stiffness of the fracture, respectively; the subscripts “n” and “s” denote the normal and shear components, respectively; the superscripts “ + ” and “–” refer to the half-spaces of either side of the fracture. The degree of displacement discontinuity is controlled by the fracture stiffness, with the limits of zero and infinity representing the cases of a free surface and a welded interface, respectively.

We use the discrete fracture network approach (Lei et al. 2017) to explicitly represent the geometrical distribution of numerous fractures in rock. The abovementioned displacement discontinuity method is employed to model the response of elastic waves at each individual fracture in the system. This allows us to capture the emergence of macroscopic wavefield properties as a result of the interaction between elastic waves and fracture populations. The validity and accuracy of our numerical model for simulating wavefield evolution in unbounded fractured media have been examined via a detailed comparison to analytical solutions and a comprehensive investigation of synthetic fracture networks (Lei and Sornette 2021a). In this paper, we additionally present an examination of the model for simulating the conversion between P and S waves when the wave propagates obliquely across a fracture (see Appendix A). In the current paper, we will use this model to study the transport and arrival behavior of plane incident waves through a natural fracture system.

### Model Setup

**Fig. 1:**
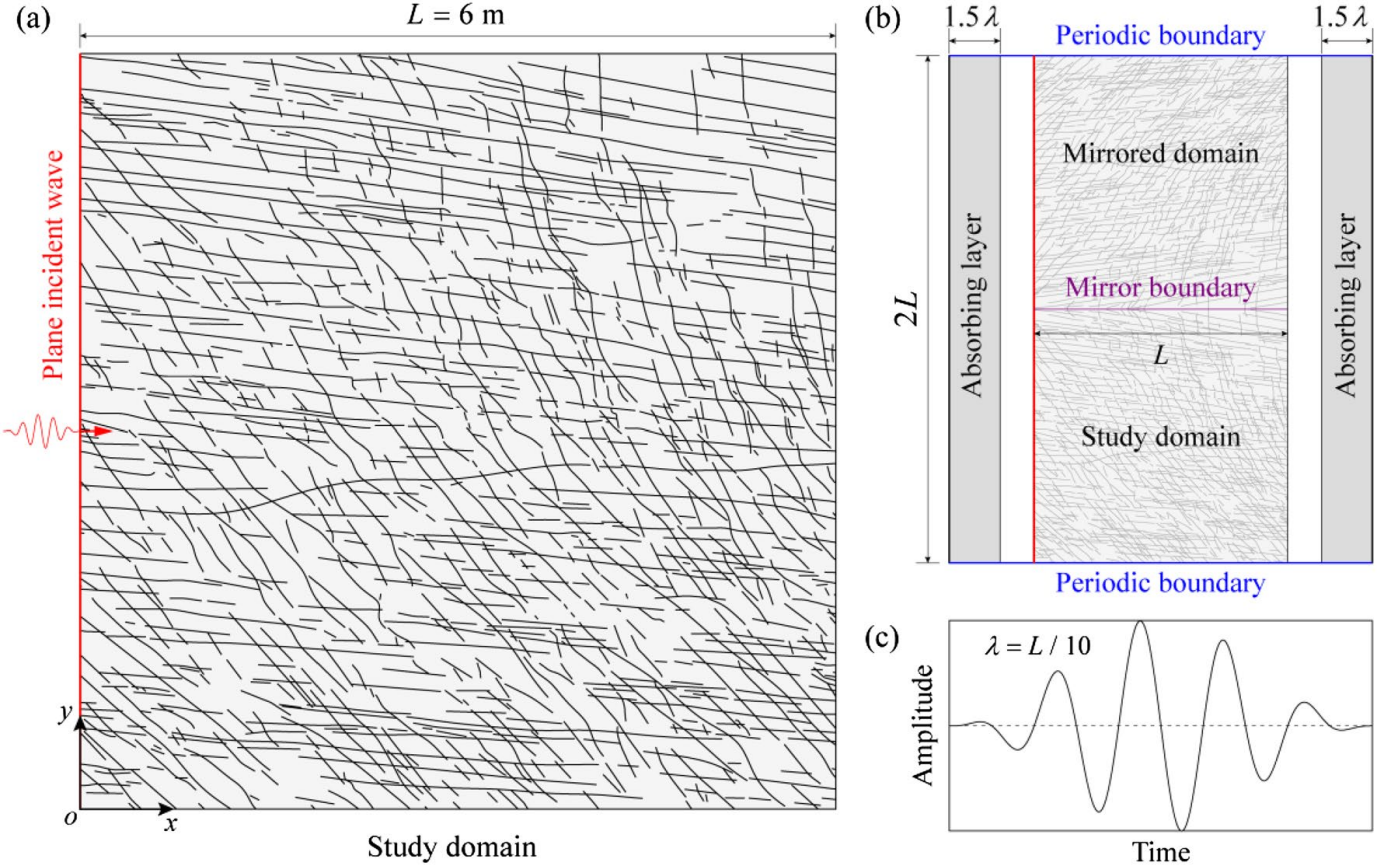
Model setup for simulating wave transport through a naturally fractured rock: **a** the natural fracture network; **b** configuration of the absorbing layers and boundary conditions; **c** wavelet of the incident wave, i.e., a five-cycle Hann-windowed tone burst

The natural fracture network used is based on the outcrop of a real fracture system in limestone mapped at Kilve on the southern margin of the Bristol Channel Basin (Belayneh et al. 2009). We extract a squared domain of size *L* = 6 m containing ~1000 fractures for numerical simulation here (Fig. 1a). In this fracture pattern, the minimum and maximum fracture lengths are *l*_min_ = 0.03 m and *l*_max_ = 4.70 m, respectively. The numerical model setup is as shown in Fig. 1b. The fracture network is placed in a 6 m × 6 m study domain, above which an auxiliary domain (embedded with exactly the same fracture network but flipped by the top of the original network) is generated via a mirroring operation. In other words, the fracture networks of the two domains are symmetrical about the mirror boundary (i.e., top of the original pattern) such that the two domains together form a unit cell (Choi et al. 2013). Then, periodic boundary conditions are imposed at the top and bottom of the cell, while absorbing layers (with a thickness of 1.5*λ* each) are attached to the left and right. Such a model configuration is designed for simulating plane wave propagation through the system. We assume typical material properties for the limestone here, as listed in Table 1 (Lama and Vutukuri 1978; Lei et al. 2015). The source line for the plane incident wave (red line in Fig. 1a and b) is located at the left side of the fracture network. A force-type signal of five-cycle Hann-windowed tone burst (Fig. 1c) is applied at the source line exciting a plane P wave with the wavelength *λ* = *L*/10 = 0.6 m, which satisfies *l*_min_ ≪ *λ* ≪ *l*_max_, such that the wavefield is considered to be in the fractal regime (Wu and Aki 1985). It should be noted that the fracture aperture is at a millimeter scale (Belayneh et al. 2006), which is much smaller than the wavelength of 0.6 m.

**Table 1:** Material properties assumed for the limestone

Properties	Value	Unit
Density, *ρ*	2700	kg/m^3^
Young’s modulus, *E*	30	GPa
Poisson’s ratio, *ν*	0.27	–
P wave velocity, *V*_P_	3726.1	m/s
S wave velocity, *V*_S_	2091.5	m/s

When P waves propagate in fractured media, various wave dynamics such as transmission, reflection, refraction, diffraction, and scattering may occur due to the presence and effect of fractures. The wave propagation behavior across a fracture of stiffness *κ* is closely related to the properties of the fracture, wave, and matrix (Pyrak-Nolte et al. 1990), which can be characterized by the dimensionless angular frequency *ῶ* = *ωZ*/*κ* with *ω* being the angular frequency of the incident wave and *Z* being the seismic impedance (i.e., the density of the rock times the phase velocity of the wave). In this study, a series of dimensionless angular frequencies *ῶ* is explored, ranging from 0.1 to 1000, with the corresponding fracture stiffness *κ* = *Zω*/*ῶ* varying from 0.3926 GP/m (for *ῶ* = 1000) to 3926 GP/m (for *ῶ* = 0.1), given that the wavelength *λ* = *L*/10. Here, we assume that fractures have an equal normal and shear stiffness for simplicity. These assigned stiffness values are designed to explore conditions associated with a wide range of overburden stress levels (or depths) (Hobday and Worthington 2012). Note that we focus on the low-amplitude wave scenario with the force-type signal imposed at the source line having a magnitude of 1000 Pa, such that the nonlinear effect of fracture deformation is negligible. The spatial domain is discretized using an unstructured grid of Delaunay triangular elements with an average element size of *l*_ele_ = *λ*/15 = 0.04 m, and the time domain is discretized using a time step controlled by a Courant–Friedrichs–Lewy (CFL) number of 0.1 (Pettit et al. 2014), i.e., the time step d*t* = *l*_ele_ / *V*_P_ × CFL = 1.07 × 10^–6^ s. The selected average element size and time step are proven to be sufficient for ensuring the simulation accuracy with an error less than 2% (Lei and Sornette 2021a). During the wave transport, P and S waves may be converted to each other (Carcione 2015). To record the wave signals (displacement in the *x* direction, i.e., same as the incident wave direction) during the simulation, a dense array of receivers (301 × 301) is evenly distributed through the study domain with a separation distance of *δ* = *L*/300 = 0.02 m.

### Characterization of First Arrival Waves

We analyze the simulation results to quantitatively investigate the wave arrival behavior in the naturally fractured rock. Wave front, defined as the leading edge of a propagating wave, is usually used to describe the first arrival of wave energies (Hamzehpour et al. 2014), i.e., the set of points (receivers) with the largest distance from the source which has the same phase (Allaei and Sahimi 2006). The wave front will keep a planar shape when the elastic plane wave propagates through a homogeneous medium (Carcione 2015). However, the wavefield will be distorted if heterogeneities are present (Åström et al. 1997; Riyanti and Herman 2005; Allaei and Sahimi 2006; Khoshhali and Hamzehpour 2015). Especially in highly heterogeneous media, the incident waves could be multiply scattered, leading to the emergence of diffusive transport lacking an apparent wave front (Mandelis 2000; Riyanti and Herman 2005; Sato and Fehler 2009; Lei and Sornette 2021a). It will be difficult to define the conventional wave front according to the wave phase since the receivers only record statistical wave signals with different oscillation patterns from the incident waves (Mandelis 2000; Hamzehpour et al. 2014). Instead of using the wave phase, the wave amplitude of a point can be alternatively used to represent wave energies passing the point (Aki and Richards 2002; Hamzehpour et al. 2014, 2016). In this regard, we use the wave amplitude to determine the leading front of wave energies to describe the arrival behavior of waves at receivers, which is defined as the front of first-arrival wave (FFAW). The FFAW consists of the points with the largest distance from the source which have the same displacement-to-amplitude ratio as

4 $$\frac{{\left| {U\left( {t_{i} ,x_{i} ,y_{i} } \right)} \right|}}{{A\left( {x_{i} ,y_{i} } \right)}} = \frac{{\left| {U^{0} \left( {t^{0} ,x_{i} ,y_{i} } \right)} \right|}}{{A^{0} \left( {x_{i} ,y_{i} } \right)}},$$

where $A^{0} \left( {x_{i} ,y_{i} } \right)$ and $A\left( {x_{i} ,y_{i} } \right)$ are the wave amplitudes recorded by a receiver $\left( {x_{i} ,y_{i} } \right)$ in homogeneous and heterogeneous media, respectively; *t*^0^ and *t*_*i*_ are the times of wave arrival at the receiver $\left( {x_{i} ,y_{i} } \right)$ in homogeneous and heterogeneous media, respectively; $U^{0} \left( {t^{0} ,x_{i} ,y_{i} } \right)$ and $U\left( {t_{i} ,x_{i} ,y_{i} } \right)$ are the receiver displacements at time *t*^0^ in the homogeneous medium and *t*_*i*_ in the heterogeneous medium, respectively. If using the same traveling time, we have

5 $$\frac{{\left| {U\left( {t_{i} ,x_{j} ,y_{j} } \right)} \right|}}{{A\left( {x_{j} ,y_{j} } \right)}} = \frac{{\left| {U\left( {t_{i} ,x_{i} ,y_{i} } \right)} \right|}}{{A\left( {x_{i} ,y_{i} } \right)}}$$

and thus determine a series of points forming the FFAW. The FFAW corresponds to the conventionally defined wave front for the wave propagating in homogeneous (or weakly heterogeneous) media, while it is more broadly used here to also characterize the leading front of a diffusive wavefield in strongly heterogeneous media with no apparent wave front. Hence, we define FFAW as a more generalized term that can be used for different wave transport regimes (e.g., normal and anomalous diffusion regimes) compared to the conventionally used wave front which is only applicable for the propagative regime.

The FFAW of the wavefield may be distorted by heterogeneities, and thus characterized by a rough profile associated with fractal and self-affine properties (Sahimi 2003; Allaei and Sahimi 2006; Khoshhali and Hamzehpour 2015). We systematically characterize the properties of the FFAW based on different approaches described as follows.

At any given time, the position of a point (i.e., a receiver) on the FFAW, i.e., its distance from the source line at *x* = 0 (Fig. 1a), is denoted as $d\left(\widetilde{t},{y}_{i}\right)$ with *y*_*i*_ being its coordinate along the *y* axis. Herein, the wave traveling time *t* is normalized to be dimensionless as $\widetilde{t}=t/{t}_{0}$, where *t*_0_ is the time of wave arrival at the right boundary in intact rock without fractures, i.e., *t*_0_ = *L*/*V*_P_. We determine $d\left(\widetilde{t},{y}_{i}\right)$ based on the seismogram recorded by each receiver. The roughness *R*($\widetilde{t}$) (Barabási and Stanley 1995) of the FFAW is defined as

6 $$R\left( {\widetilde{t}} \right) = \sqrt {\frac{1}{{N_{y} }}\sum\limits_{i = 1}^{{N_{y} }} {\left[ {d\left( {\widetilde{t},y_{i} } \right) - \overline{{d\left( {\widetilde{t}} \right)}} } \right]}^{2} } ,$$

where $\overline{{d\left( {\tilde{t}} \right)}}$ is the average distance of points on the FFAW from the source line at the dimensionless time $\widetilde{t}$; *N*_*y*_ is the number of receivers (*N*_*y*_ = 301 in our current model).

To evaluate the fractal characteristic of such a non-planar FFAW, the fractal dimension *D* (Falconer 2003) is derived as

7 $$D = 1 + \frac{{\log_{10} \left( {L_{{{\text{FFAW}}}} \left( {\delta l} \right)} \right)}}{{\log_{10} \left( {1/\delta l} \right)}},$$

where *δl* is the measurement scale and *L*_FFAW_(*δl*) is the length of a FFAW measured by the scale *δl*. For a given *δl*, the length of FFAW is measured in some way ignoring irregularities of size less than *δl*. We analyze a range of *δl* values and calculate the corresponding *L*_FFAW_. In our numerical model, the distance of adjacent receivers is 0.02 m. Thus, the scanned *δl* ranges from 0.02 m to the domain size of 6 m.

Furthermore, the self-affine shape of the FFAW can be characterized by a roughness exponent *α*, which can be derived from the second-order front–front correlation function *C*(Δ) (Barabási and Stanley 1995; Allaei and Sahimi 2006)

8 $$C(\Delta ) = \left\langle {\left[ {d(y) - d(y + \Delta )} \right]^{2} } \right\rangle ,$$

where Δ is the distance of two points on the surface and the angle bracket means the average over each value of Δ. To evaluate the self-affine property of a FFAW of simulated wavefield, Δ ranges from 0.02 m (i.e., the separation distance of receivers) to 5.98 m (i.e., *L*–0.02 m) while *y* ranges from 0 m to (*L* − Δ). For a self-affine rough front, the correlation function obeys (Allaei and Sahimi 2006; Khoshhali and Hamzehpour 2015)

9 $$C(\Delta )\sim \Delta^{2\alpha } ,$$

where *α* is the roughness exponent. Note that the roughness *R* is an estimation of the overall fluctuation (i.e., the standard deviation) of a FFAW, while the roughness exponent *α* generally represents the local volatility of a FFAW (Mandelbrot 1985).

## Results

### Spatiotemporal Evolution of the Wavefield

**Fig. 2:**
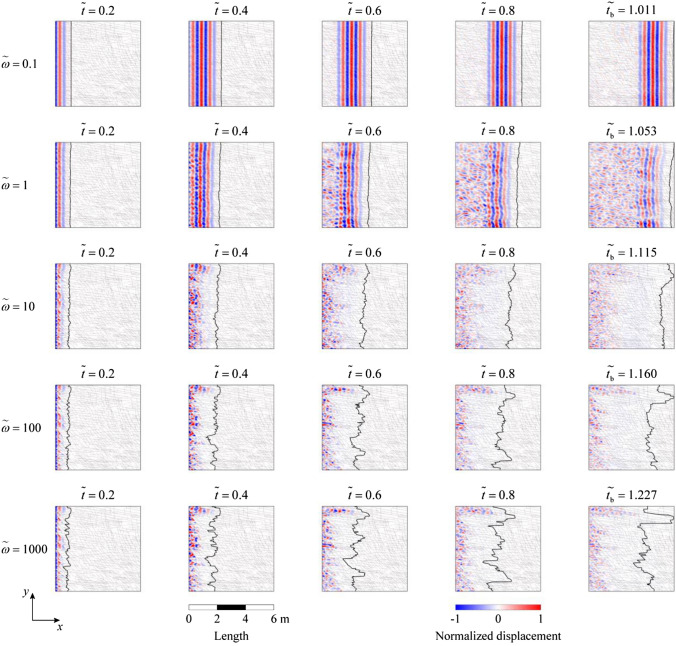
Spatiotemporal evolution of the wavefield (normalized displacement in the *x* direction) together with the FFAW indicated by the *solid black line* for different dimensionless angular frequencies *ῶ* at different dimensionless times $\widetilde{t}$ = 0.2, 0.4, 0.6, 0.8, and the breakthrough time ${\widetilde{t}}_{\mathrm{b}}$. Note that different *ῶ* cases have different ${\widetilde{t}}_{\mathrm{b}}$ values

**Fig. 3:**
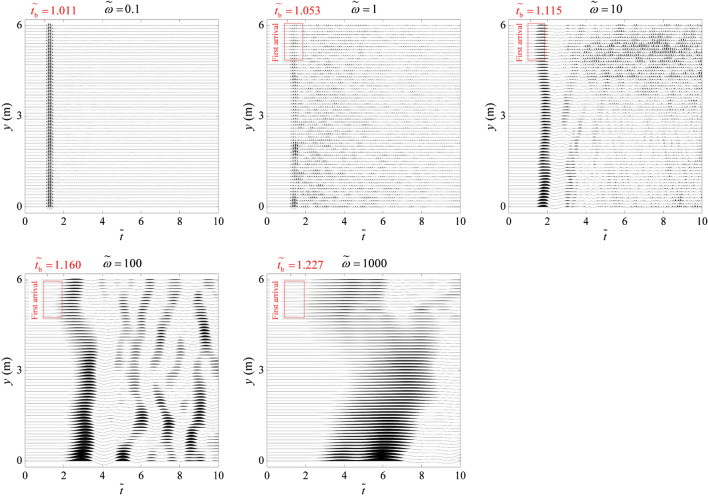
Seismograms (normalized displacement in the *x* direction) recorded by the receivers at the right boundary of the fracture network for the cases of different dimensionless angular frequencies *ῶ* = 0.1, 1, 10, 100, and 1000

**Fig. 4:**
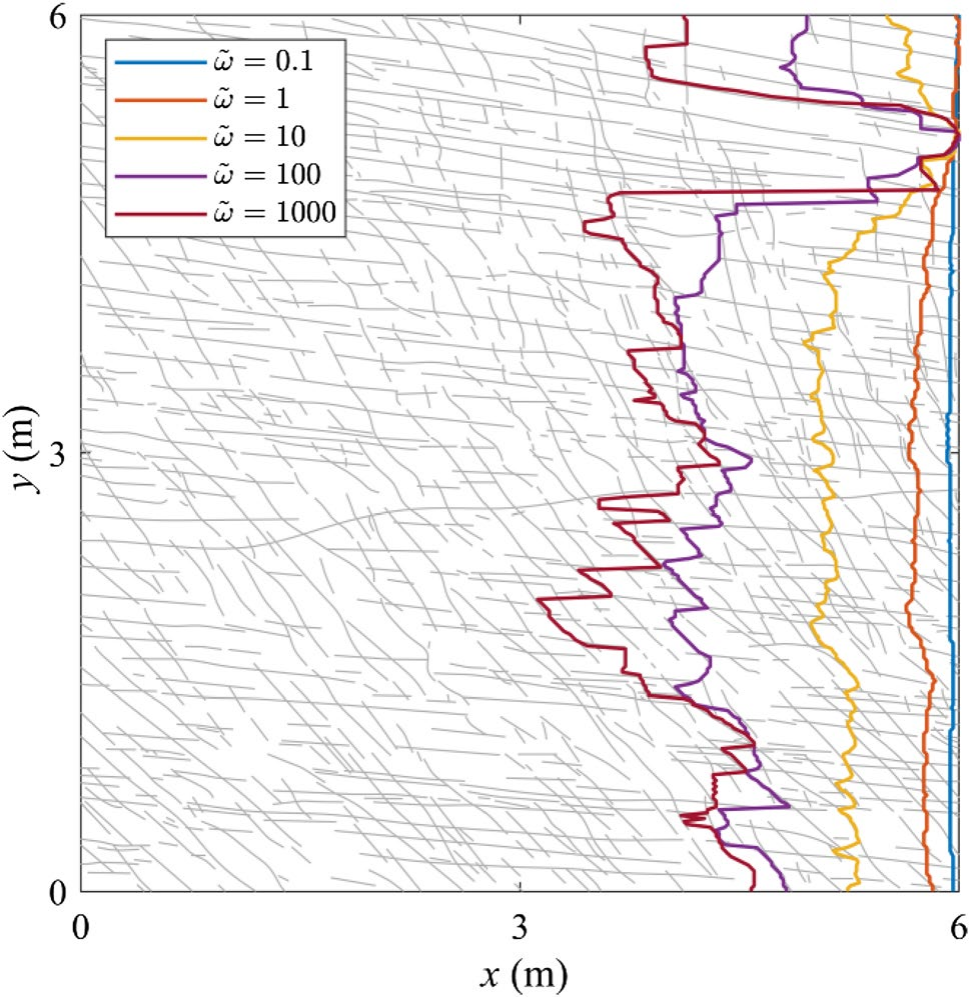
FFAWs at the breakthrough time for different dimensionless angular frequencies *ῶ* = 0.1, 1, 10, 100, and 1000

**Fig. 5:**
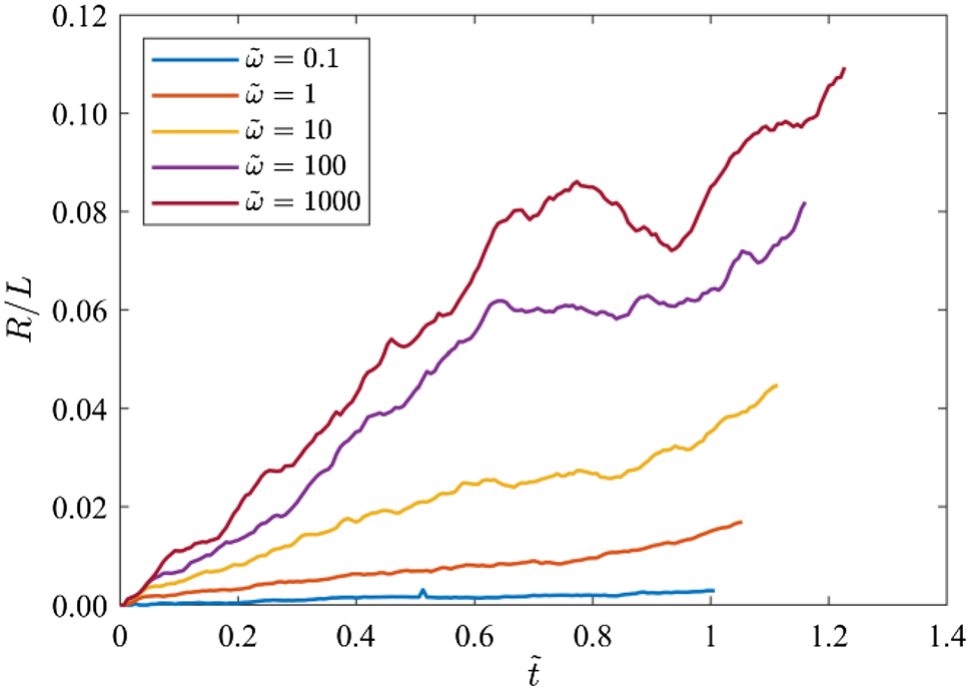
Temporal evolution of the FFAW roughness *R* as a function of the dimensionless time $\widetilde{t}$ for different dimensionless angular frequencies *ῶ* = 0.1, 1, 10, 100, and 1000

Figure 2 shows the spatiotemporal evolution of the elastic wavefield (illustrated by the displacement in the *x* direction normalized by the displacement amplitude of the incident wave) together with its FFAW for different dimensionless angular frequencies *ῶ* at different dimensionless times $\widetilde{t}$ = 0.2, 0.4, 0.6, 0.8. We also plot the wavefield and FFAW pattern for the breakthrough time ${\widetilde{t}}_{\mathrm{b}}$, at which the wave energy for the first time reaches the right boundary (note that different *ῶ* cases have different ${\widetilde{t}}_{\mathrm{b}}$ values). Seismograms recorded by the receivers at the right boundary are given in Fig. 3 with ${\widetilde{t}}_{\mathrm{b}}$ indicated for different *ῶ*. We also compare their FFAW patterns at ${\widetilde{t}}_{\mathrm{b}}$ in Fig. 4. Furthermore, Fig. 5 shows the variation of the FFAW roughness *R* (normalized by *L*) as a function of the dimensionless time $\widetilde{t}$ for different *ῶ* cases.

As shown in Fig. 2, when the dimensionless angular frequency is low, i.e., *ῶ* = 0.1, fractures have a relatively high stiffness and thus exert little impact on the wave transport. In general, the wavefield is in a propagative mode with little or limited energy scattered. The FFAW keeps its planar shape in the early stage ($\widetilde{t}$ ≤ 0.6), but tends to be slightly distorted as the wave propagates further ($\widetilde{t}$ ≥ 0.8). When the wave breakthrough occurs at ${\widetilde{t}}_{\mathrm{b}}$ = 1.011 (Figs. 2 and 3), it first penetrates through the upper region of the right boundary (Figs. 2, 3, 4) and the rest arrives soon after, exhibiting in general a synchronous arrival behavior. The FFAW roughness grows slowly as the wave propagates (Fig. 5).

For *ῶ* = 1, fractures become more visible to the wave due to the reduced fracture stiffness. Consequently, the wavefield is significantly scattered by fractures, resulting in the emergence of diffusive coda waves. The FFAW exhibits a slightly rough profile while some scattered coda waves are also produced behind the frontal wave in early time (e.g., $\widetilde{t}$ ≤ 0.4) (Fig. 2). As the wave travels further (e.g., $\widetilde{t}$ ≥ 0.6), more high-frequency components of the wave energy are transformed into coda waves as a result of reflection, diffraction, and scattering by fractures (Figs. 2 and 3), such that the FFAW becomes more distorted and associated with an increased roughness (Fig. 5). Waves break through the fractured rock at ${\widetilde{t}}_{\mathrm{b}}$ = 1.053 with the first penetration occurring at the upper region of the right boundary (Fig. 4). By comparing the wavefields at ${\widetilde{t}}_{\mathrm{b}}$, it can be observed that the asynchronous arrival phenomenon of wave energies becomes more significant than the case of *ῶ* = 0.1.

For larger values of *ῶ* (i.e., *ῶ* ≥ 10), the wavefield becomes more diffusive in the early stage ($\widetilde{t}$ ≤ 0.4) due to the reflection and scattering by fractures of small stiffness (Fig. 2). Fractures that interconnect with each other (especially those longer than the wavelength) act as strong reflection barriers to waves. Only very low frequency components of wave energy may break through the fracture network, whereas the high-frequency contents are almost completely trapped (Fig. 2). As a result, the wavefield is highly distorted associated with a rough FFAW (Fig. 4) and more coda waves (Fig. 3). Only a small portion of wave energies (low frequency contents) can penetrate through the fracture network and finally arrives at the right boundary (see ${\widetilde{t}}_{\mathrm{b}}$ = 1.115, 1.160, and 1.227 for *ῶ* = 10, 100, and 1000, respectively, in Fig. 3). The first-arrival regions of these three cases are also located in the upper region of the right boundary, similar to *ῶ* = 1 (Fig. 4). It can also be observed that the asynchronous arrival behavior of waves is more significant (Figs. 3 and 4) and the FFAW roughness grows more rapidly, with the increase of *ῶ* (Fig. 5).

### Characterization of Wave Arrival Behavior

**Fig. 6:**
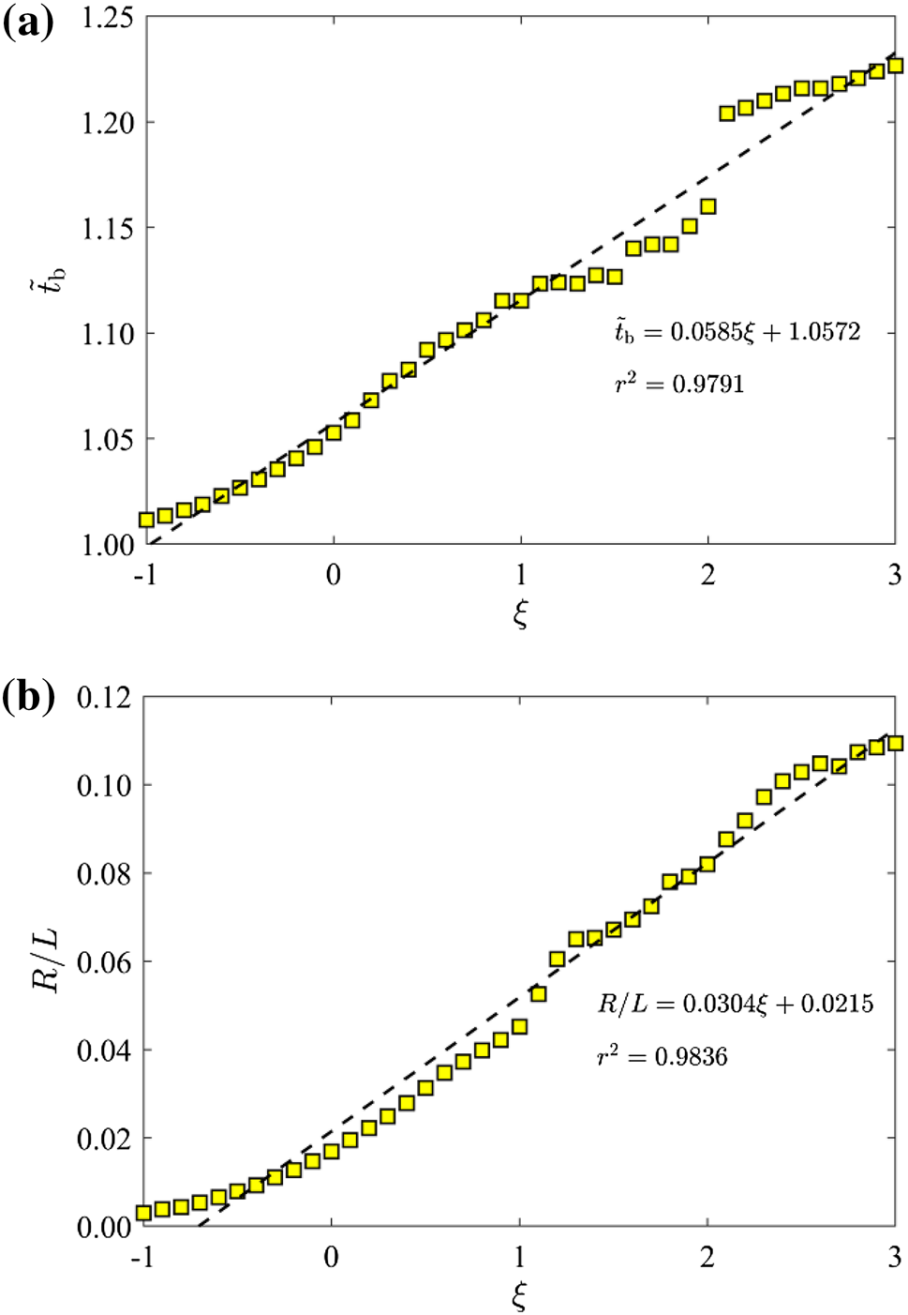
**a** The breakthrough time ${\widetilde{t}}_{\mathrm{b}}$ and **b** the roughness *R* of the FFAW at ${\widetilde{t}}_{\mathrm{b}}$ as a function of the exponent *ξ* (with *ῶ* = 10^*ξ*^). *Dashed lines* indicate the fitting curves

In general, as *ῶ* increases, more high-frequency wave components are transformed into coda waves and less wave energies can travel through the fracture network (Fig. 2). Correspondingly, the breakthrough time ${\widetilde{t}}_{\mathrm{b}}$ tends to increase (Fig. 3) and the FFAW roughness grows more rapidly and significantly (Figs. 4 and 5). To further investigate the arrival behavior of waves in this natural fracture network, we conduct a series of numerical simulations with the exponent *ξ* (for *ῶ* = 10^*ξ*^) ranging from -1 to 3 at a step of 0.1. As mentioned above, fractures with a smaller stiffness (i.e., a higher dimensionless angular frequency) exert a more profound impact on wave transport. One consequence is that the breakthrough time ${\widetilde{t}}_{\mathrm{b}}$ linearly increases as a function of *ξ* (with the coefficient of determination *r*^2^ of the fitting larger than 0.97), as shown in Fig. 6a. Another phenomenon is that the FFAW at the breakthrough time becomes rougher with an increased *ῶ* (i.e., a reduced fracture stiffness), and the roughness *R* shows a positive linear relationship with *ξ* (the coefficient of determination *r*^2^ is 0.98 for the fitting) (Fig. 6b). A rough front usually exhibits a pattern that can be characterized by fractal and self-affine properties (Sahimi 2003; Allaei and Sahimi 2006; Khoshhali and Hamzehpour 2015). In the following, we further characterize the wave arrival behavior by analyzing the fractal and self-affine characteristics of the FFAW at the breakthrough point.

**Fig. 7:**
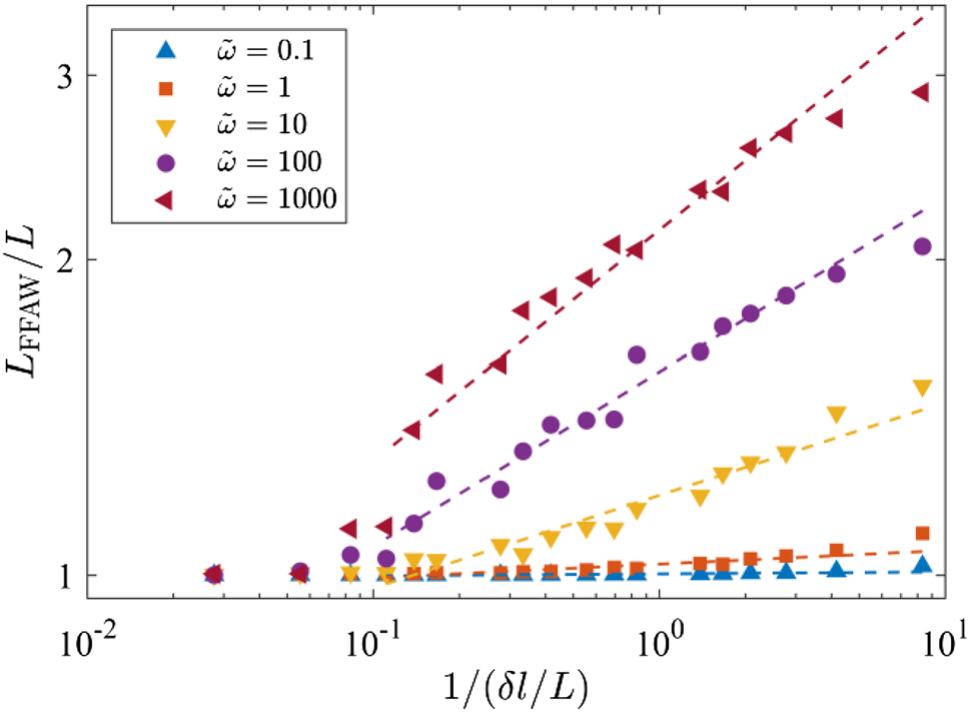
Measured FFAW length *L*_FFAW_ at the breakthrough time ${\widetilde{t}}_{\mathrm{b}}$ as a function of the reciprocal measurement scale *δl* for different dimensionless angular frequencies *ῶ* = 0.1, 1, 10, 100, and 1000. The *dashed line* for each case indicates the fitting curve, whose slope is used to calculate the fractal dimension *D*

**Fig. 8:**
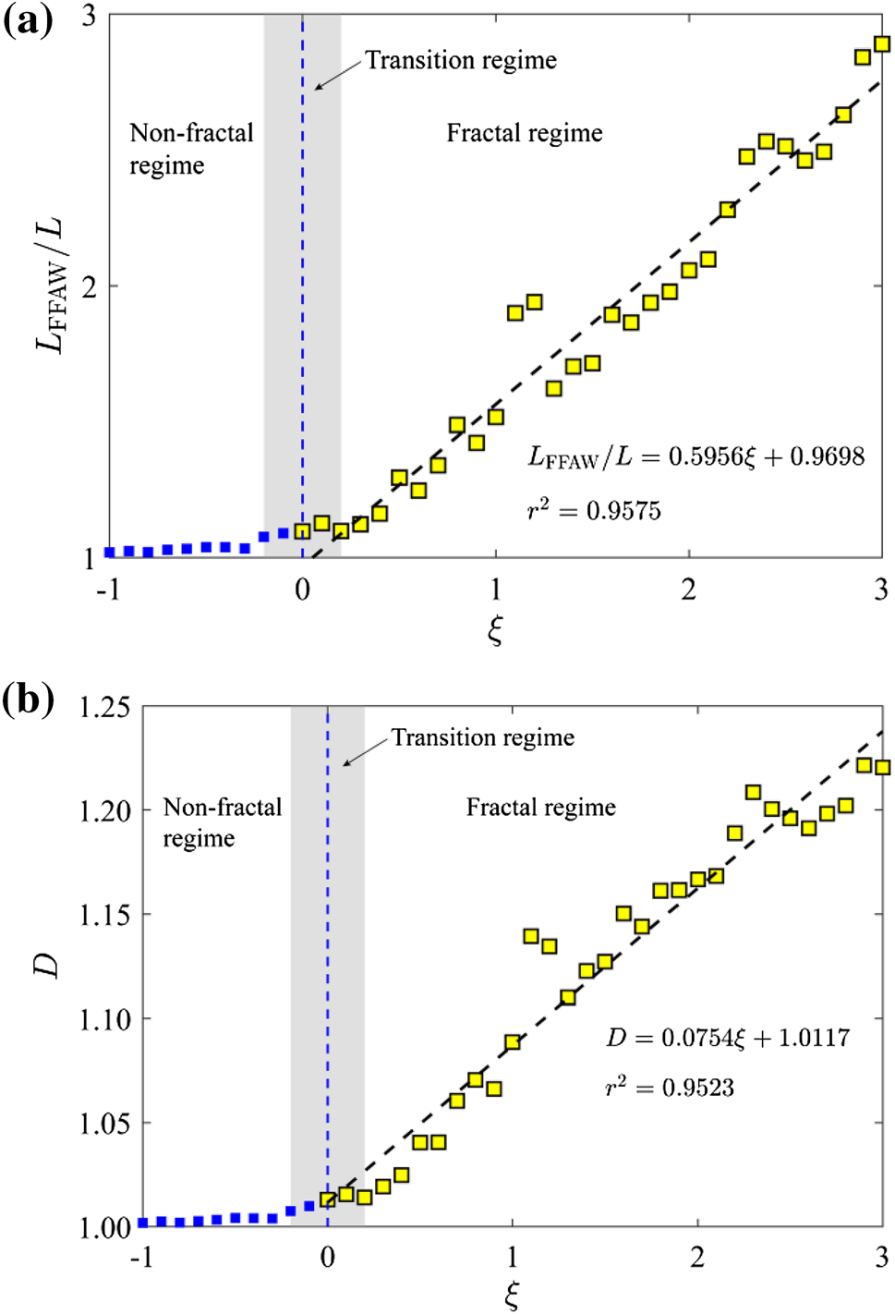
**a** Measured *L*_FFAW_ for *δl* = 0.02 m and **b** the fractal dimension *D* of the FFAW at the breakthrough time ${\widetilde{t}}_{\mathrm{b}}$ as a function of the exponent *ξ*. *Dashed lines* indicate the fitting curve

As stated in Sect. 2.3, the fractal dimension *D* can be derived from the relationship between the FFAW length *L*_FFAW_ and the measurement scale *δl*. Figure 7 gives the length of FFAW at the breakthrough time as a function of 1/*δl* (i.e., the reciprocal of measurement scale) for the cases of *ῶ* = 0.1, 1, 10, 100, and 1000. Note that *L*_FFAW_ and *δl* are both normalized by the fracture network size of *L* = 6 m in Fig. 7. The measured length of FFAW increases with 1/*δl* linearly on the log–log plot. By fitting a straight line to the data (see the dashed line in Fig. 7), we can calculate the fractal dimension *D* using Eq. (7). We also analyze the variation of the measured FFAW length at the breakthrough time (Fig. 8a) and the associated fractal dimension *D* (Fig. 8b) as a function of *ξ*. As shown in Fig. 8, two scaling regimes for *D* and *L*_FFAW_ versus *ξ* can be identified with a transition occurring at *ῶ*_c_ ≈ 1.0, where *ῶ*_c_ is defined as the critical dimensionless angular frequency (the corresponding critical exponent is *ξ*_c_ ≈ 0). When *ῶ* is small (*ῶ* < *ῶ*_c_), *L*_FFAW_ keeps a value around the fracture network size *L* (i.e., consistent with its planar shape) and *D* is close to 1.0, indicating that the FFAW is non-fractal. For *ῶ* ≥ *ῶ*_c_, the two parameters *L*_FFAW_ and *D* both linearly increase with *ῶ*, such that the fractality of the FFAW becomes more and more evident. Thus, we classify the FFAW behavior into two different regimes: a non-fractal regime for *ῶ* < *ῶ*_c_ and a fractal regime for *ῶ* ≥ *ῶ*_c_. By referring to Figs. 2 and 3, it is considered that this transition may be related to the transition of the wavefield from a single scattering-dominated regime to a multiple scattering-dominated one.

**Fig. 9:**
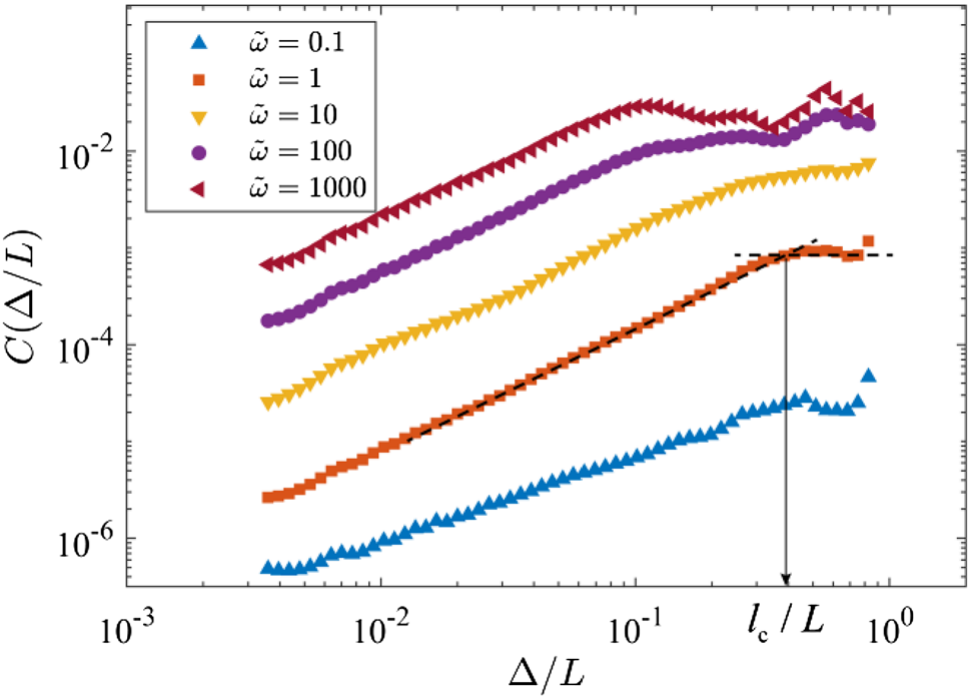
Correlation function *C*(Δ/*L*) of the FFAW at the breakthrough time as a function of Δ/*L* for different dimensionless angular frequencies *ῶ* = 0.1, 1, 10, 100, and 1000. *Dashed lines* are the fitting results of the two segments in *C*(Δ/*L*) and their intersection points are used to derive the correlation length *l*_c_

**Fig. 10:**
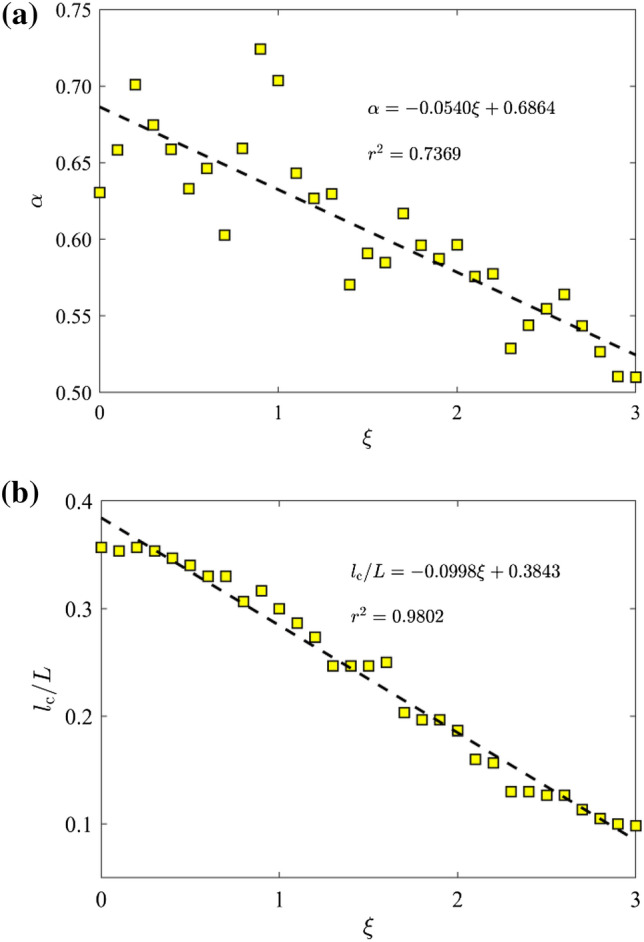
**a** The roughness exponent *α* and **b** the correlation length *l*_c_ of the FFAW at the breakthrough time as a function of the exponent *ξ* in the fractal regime (with *ῶ* ≥ *ῶ*_c_ ≈ 1.0 or *ξ* ≥ *ξ*_c_ ≈ 0). *Dashed lines* indicate the fitting curves

The self-affine characteristic of a fractal profile is usually represented by the roughness exponent *α* (Allaei and Sahimi 2006). Figure 9 displays the correlation function *C*(Δ/*L*) of the FFAW at the breakthrough time as a function of Δ/*L* for the cases of *ῶ* = 0.1, 1, 10, 100, and 1000. On the log–log graph, the correlation function first linearly increases with Δ/*L* when Δ/*L* is small, i.e., a power-law scaling between *C*(Δ/*L*) and Δ/*L*, and then *C*(Δ/*L*) attempts to plateau when Δ/*L* is large. The slope of the fitting line on the log–log plot corresponds to 2*α* as expressed in Eq. (9), allowing us to derive the roughness exponent *α*. Figure 10a gives the determined *α* values as a function of *ξ* in the fractal regime (i.e., *ῶ* ≥ *ῶ*_c_ ≈ 1.0) for which a self-affine curve must first have a fractal structure (Mandelbrot 1985; Falconer 2003). In general, *α* linearly decreases as a function of *ξ*. The turnover of the correlation function *C*(Δ/*L*) versus Δ/*L* is characterized by the correlation length *l*_c_ (Khoshhali and Hamzehpour 2015), as shown in Fig. 9. Figure 10b shows that *l*_c_/*L* decreases linearly as a function of *ξ*. The decreasing relationship manifests that if *ξ* increases, the FFAW’s self-affinity is associated with a shorter correlation length *l*_c_ and the FFAW profile becomes rougher.

## Discussion

**Fig. 11:**
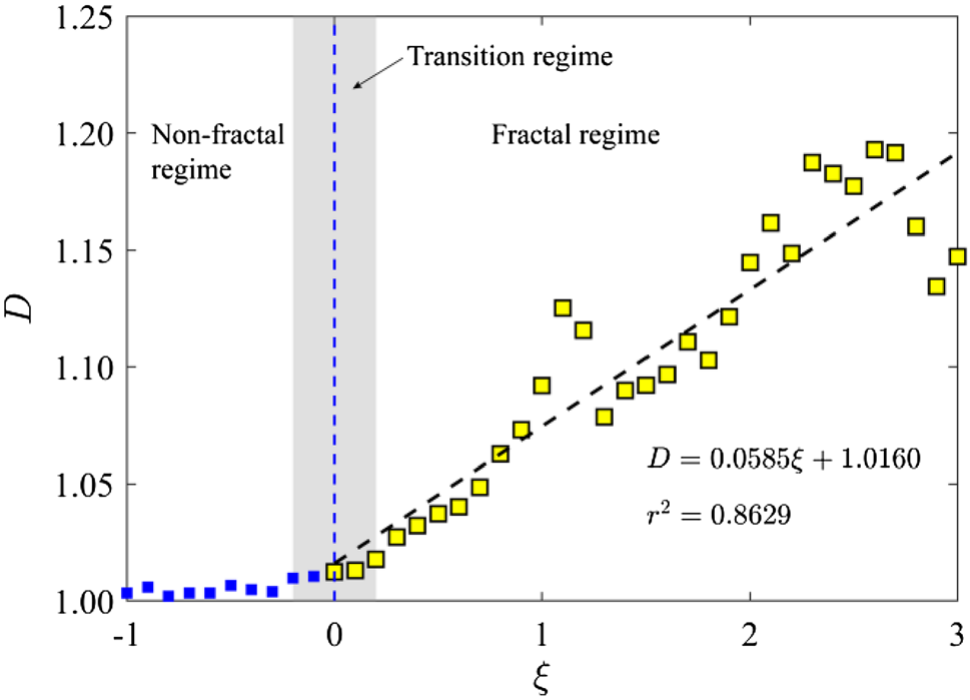
Fractal dimension *D* of the FFAW at the breakthrough time as a function of the exponent *ξ* computed using the same model as in Fig. 2 but with an incident plane wave exciting from the right boundary of the fracture network. The *dashed line* indicates the fitting curve. Same with Fig. 9b, *ῶ*_c_ ≈ 1.0 (*ξ*_c_ ≈ 0) defines a transition from a non-fractal regime to a fractal one

**Fig. 12:**
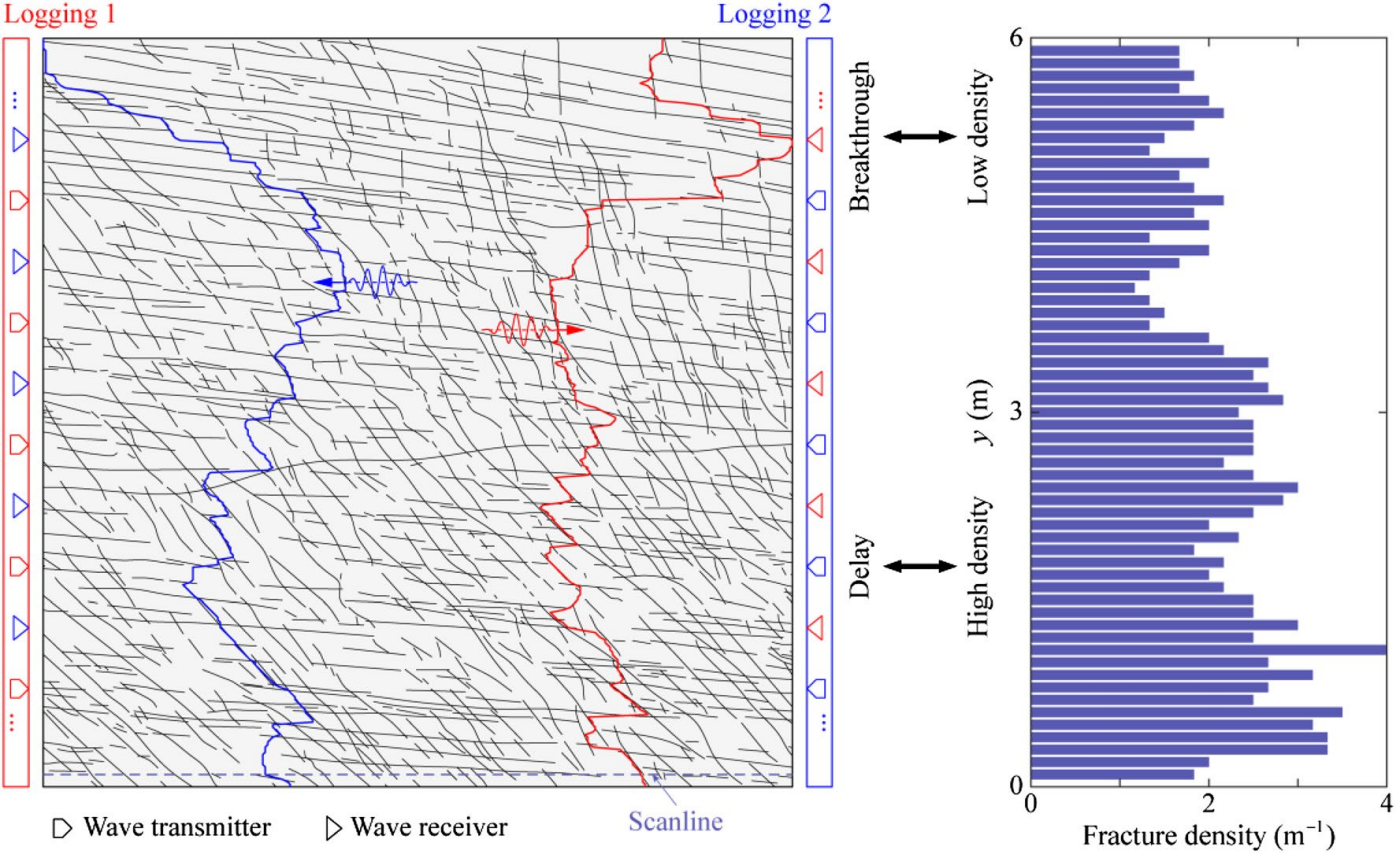
The correspondence between wave breakthrough/delay and fracture density. The left FFAW (in *blue*) is for the scenario with a plane incident wave exciting from the right boundary and the right FFAW (in *red*) is for the scenario with a plane incident wave exciting from the left boundary. Both simulations have a dimensionless angular frequency of *ῶ* = 100 (color figure online)

In this paper, we studied the elastic wave transport and arrival behavior in a naturally fractured rock associated with complex fracture network geometries. To better understand the relationship between the wave arrival behavior and the distribution of the natural fracture network, we further simulate the wave transport for the scenario where an incident plane wave is excited from the right boundary of the fracture network. We explore the same values of dimensionless angular frequency *ῶ* = 10^*ξ*^ with *ξ* ranging from -1 to 3 at a step of 0.1. The corresponding fractal dimension *D* of the FFAW at the breakthrough point is shown in Fig. 11. Similar to Fig. 8b, a transition occurs at *ῶ*_c_ ≈ 1.0 (*ξ*_c_ ≈ 0) from a non-fractal regime to a fractal one. As we selected a wavelength of *λ* = *L*/10 = 0.6 m, satisfying *l*_min_ ≪ *λ* ≪ *l*_max_, the wavefield is expected to be in a fractal regime (Wu and Aki 1985). However, the FFAW at the breakthrough time only exhibits a significant fractal characteristic for when the dimensionless angular frequency *ῶ* ≥ *ῶ*_c_ ≈ 1.0. This is attributed to the little or limited impact of fractures on the wavefield when *ῶ* < *ῶ*_c_. In the fractal regime, the fractal dimension *D* of the FFAW at the breakthrough time has a linearly positive relationship to the exponent *ξ* (Figs. 8b and 11). We select *ῶ* = 100 as an example and show the FFAWs at the breakthrough time for the waves traveling along two different directions (i.e., from left to right or vice versa) in Fig. 12. The FFAW (in blue) of the right-to-left scenario shows an evident breakthrough region at the upper left boundary of the fracture network, which is similar to the FFAW (in red) of the left-to-right scenario. If an array of scanlines along the *x* direction is setup evenly through the fracture network, the number of intersection points between each scanline and fractures can be calculated, which can serve as an indicator of the fracture density (i.e., inverse of the fracture spacing). Figure 12 shows the fracture density measured by each scanline, where the upper region of the fracture network has a smaller density while the lower region has a higher density. Thus, we may infer that the region where wave breakthrough occurs is associated with a low fracture density while the waves are more delayed or trapped in regions with a high fracture density. This is understandable because more fractures would lead to more scattering and attenuation of the wavefield. Consequently, the wave arrival properties exhibit frequency independency to the dimensionless angular frequency in the non-fractal regime (*ῶ* < *ῶ*_c_) but begins to reflect the geometrical characteristics of a natural fracture network in the fractal regime (*ῶ* ≥ *ῶ*_c_) as *ῶ* increases. Such a correspondence between the wave breakthrough with the fracture density may have useful implications for characterizing the geometrical distributions of natural fracture systems (especially those in near-surface dry rocks). For example, one may install two loggings (loggings 1 and 2 as shown in Fig. 12) on the opposite boundaries of a fractured rock volume and excite plane waves from the logging one by one. A series of frequencies may be tested to cover the non-fractal and fractal regimes. Afterward, the captured first-arrival wave signals may be used to infer the fracture density and/or stiffness. However, to systematically establish the relationship between fracture network properties and wave arrival parameters, further investigations are needed to analyze synthetic fracture networks (Lei et al. 2017) and to test more cases of natural fracture networks (Odling 1997; Myers and Aydin 2004).

**Fig. 13:**
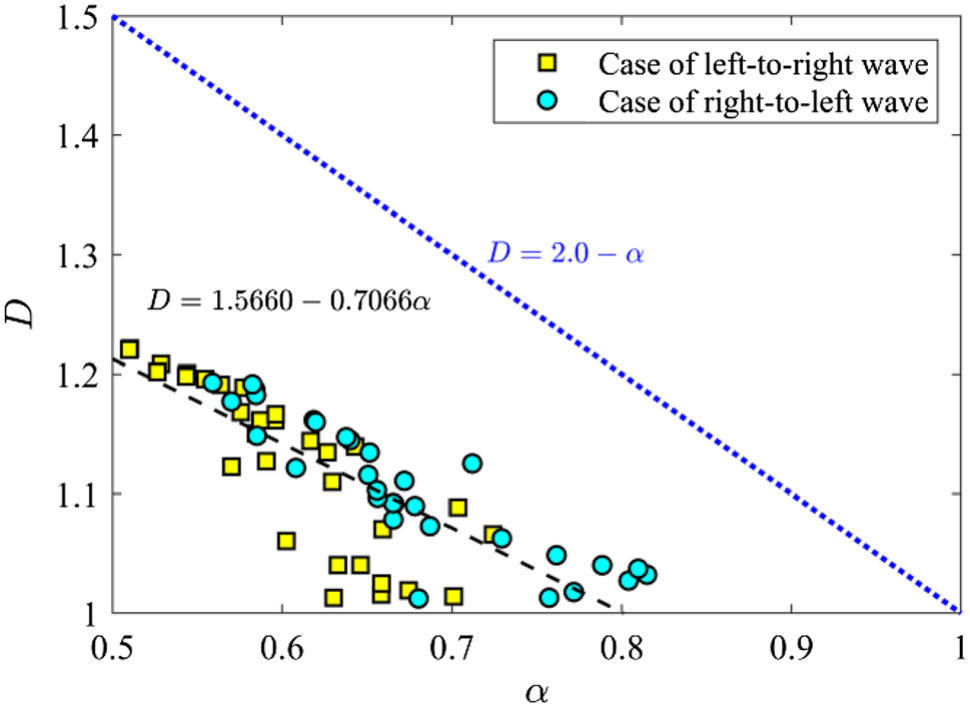
Variation of the fractal dimension *D* as a function of the roughness exponent *α* within the fractal regime (*ῶ* ≥ *ῶ*_c_ ≈ 1.0). The *dashed black line* indicates the fitting result. The *dot blue line* stands for *D* = 2.0 − *α*, representing the linear relationship between *D* and *α* anticipated for a fractal Brownian scenario

A fractal system usually exhibits a self-similarity or self-affinity characteristic (Falconer 2003). In our simulation, we found that the FFAW has a self-affine structure in the fractal regime (*ῶ* ≥ *ῶ*_c_). We further analyze the relationship between self-affine and fractal parameters of the FFAW at the breakthrough time in Fig. 13, where the fractal dimension *D* in general follows a linear relationship with *α* as *D* = 1.57 – 0.71*α*. The linear relationship herein is different from the one for a fractional Brownian curve generated by a Gaussian process (Kroese and Botev 2015), which obeys *D* = 2 − *α* (Falconer 2003). This suggests that the FFAW in the fracture network is not produced by a purely random process, but dependent on the distribution and properties of the natural fracture network, which are not Gaussian (Lei et al. 2014, 2015). We would like to emphasize that these fractal characteristics of FFAW are emergent properties arising from the complex interaction of elastic waves and numerous fractures (e.g., multiple reflection and scattering processes) in the system across spatiotemporal scales, which cannot be predicted by analytical solutions of wave propagation across a single fracture. This mechanism also explains the emergence of qualitatively different macroscopic regimes of elastic wavefields in fractured media, ranging from propagation to diffusion and to localization/delocalization, as illustrated in Fig. 2 of the current paper as well as in our previous work (Lei and Sornette 2021a, b). This wavefield phase transition underlies the observed transition of FFAW properties from a non-fractal to a fractal regime. It should be noted that our current research focused on the heterogeneity related to fractures and did not consider the heterogeneity of rock materials, of which the statistical properties have been revealed to also affect the structure of wave front (Allaei and Sahimi 2006; Hamzehpour et al. 2016). It would be of great interest to study the competing roles of the fracture network distribution and rock matrix heterogeneity in the wavefield evolution, which will be explored in our follow-up research.

In the current study, we explored the linkage between elastic wave arrival behavior and fracture network distribution, which may have important implications for geophysical characterization of subsurface fracture systems in rock. We also identify a few aspects to extend in the future to make our research more relevant to rock engineering applications. First, natural fractures in deep subsurface rocks are, in general, under saturated conditions which could cause intrinsic attenuation of seismic waves, for which the viscous damping effect due to the presence of fluid will need to be included in the displacement discontinuity model (Pyrak-Nolte et al. 1990). Second, when large-amplitude stress waves (e.g., generated by blasts or earthquakes) propagate through fractured rocks, the effects of nonlinear deformational behavior and stress-dependent stiffness of fractures may become important, for which the Barton–Bandis’ joint model is recommended to realistically reproduce the nonlinear fracture deformational behavior (Bandis et al. 1983; Zhao et al. 2008; Lei and Barton 2022).

## Conclusion

In this paper, a series of 2D numerical simulations of plane wave propagation across a naturally fractured rock was conducted to investigate the wave transport and arrival behavior. The numerical results revealed that the dimensionless angular frequency *ῶ* plays a crucial role in governing the wave transport processes and arrival properties in fractured rock. The main conclusions are given as follows. First, the spatiotemporal evolution of the wavefield showed that, as *ῶ* increases, more wave energy becomes scattered and trapped in the system with reduced wave energy penetrating through the fracture network. The breakthrough time ${\widetilde{t}}_{\mathrm{b}}$ and the roughness *R* of the FFAW both linearly increases as a function of the exponent *ξ* of the dimensionless angular frequency (*ῶ* = 10^*ξ*^). Consequently, the asynchronous arrival phenomenon of wave energies becomes more significant with an increased *ῶ*. Second, the fractal dimension *D* of the FFAW at the breakthrough time obeys a two-branch linear dependence on *ῶ* with the transition occurring at the critical frequency *ῶ*_c_ ≈ 1.0: for *ῶ* < *ῶ*_c_, *D* is slightly larger than unity and independent of *ῶ*; as *ῶ* exceeds *ῶ*_c_, *D* linearly increases as a function of the exponent *ξ*. In addition, within the non-fractal regime (*ῶ* < *ῶ*_c_), the wave arrival behavior exhibits an independency on *ῶ*; however, in the fractal regime (*ῶ* ≥ *ῶ*_c_), the geometrical characteristic of a natural fracture network has a profound impact on the wave arrival behavior. In the fractal regime, the roughness exponent *α* and the correlation length *l*_c_ of the FFAW both linearly decrease as a function of *ξ*. Furthermore, the wave breakthrough occurs in the region of a relatively low fracture density while the region of a relatively high fracture density tends to accommodate delayed arrivals. These findings in our paper have important implications for understanding the wave transport processes and phenomena in fractured crustal rocks as well as characterizing the geometrical distribution of natural fracture systems in geological media.

## Figures and Tables

**Fig. 14:**
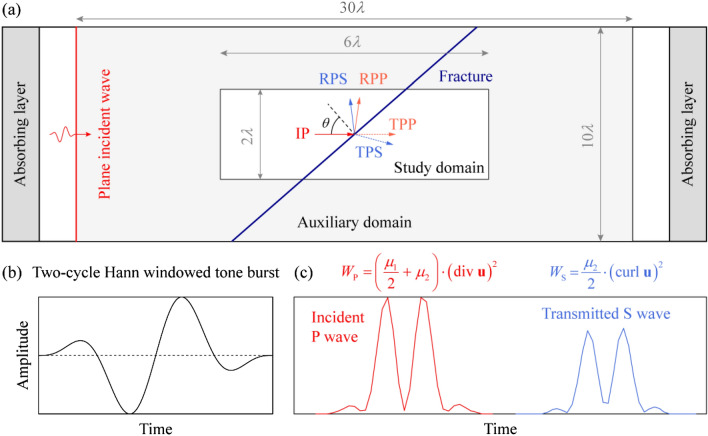
Numerical simulation of wave propagation obliquely across an infinite fracture: **a** schematic of the model setup; **b** wavelet of the incident wave, i.e., a two-cycle Hann-windowed tone burst; **c** signals of *W*_P_ (energy density for incident P wave) and *W*_S_ (energy density for transmitted S wave)

**Fig. 15:**
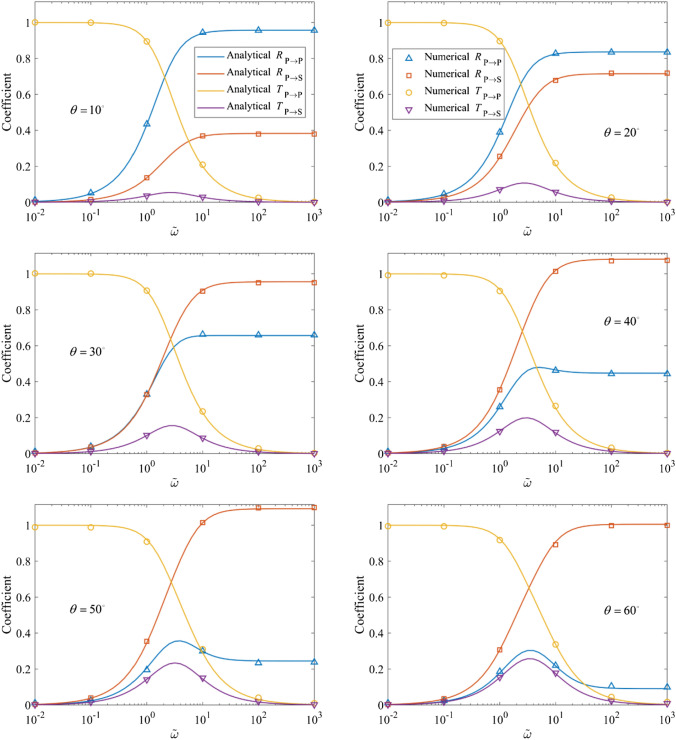
Reflection and transmission coefficients for oblique P wave propagation across an infinite fracture with an incidence angle of 10°, 20°, 30°, 40°, 50°, and 60°. The markers denote the results of numerical simulations and the solid lines denote the analytical solutions

**Fig. 16:**
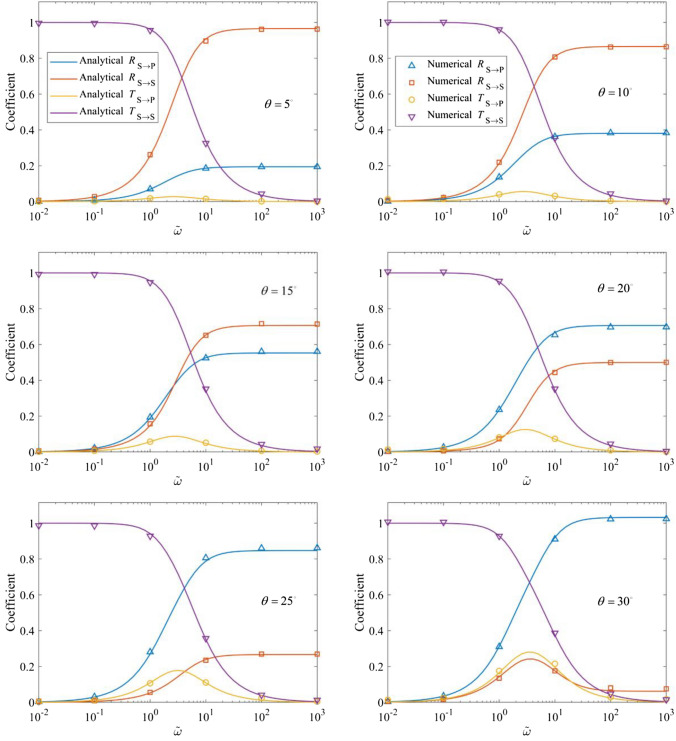
Reflection and transmission coefficients for oblique S wave propagation across an infinite fracture with an incidence angle of 5°, 10°, 15°, 20°, 25°, and 30°. The markers denote the results of numerical simulations and the solid lines denote the analytical solutions

## Data Availability

The authors declare that the data supporting the findings of this study are available within the article.

## Appendix A: Validation of Oblique Wave Propagation Across an Infinite Fracture

Our model described in Sect. 2 has been validated against the analytical solutions in our previous work for the reflection and transmission of elastic wave propagating normal at an infinite single fracture as well as for the wave scattering at a finite-sized single fracture (Lei and Sornette 2021a). Here, we include an additional validation of our model for simulating the conversion between P and S waves when obliquely propagating across an infinite fracture (Pyrak-Nolte et al. 1990). In our validation test (Fig. 14a), a single fracture is located at the middle of the domain with an inclination angle *θ* relative to the *y*-direction, equal to the incidence angle of the incident wave. The study domain is surrounded by an auxiliary domain to eliminate the boundary effect. The left and right boundaries of the model are each attached to an absorbing layer, while the top and bottom boundaries are connected via a periodic condition. The spatial domain of the model is meshed using an unstructured grid of Delaunay triangular elements with an average element size of *λ*/15, and the time domain is discretized using a time step controlled by a CFL number of 0.1. A force-type signal of two-cycle Hann-windowed tone burst (Fig. 14b) is applied at the source line (red line in Fig. 14a) to excite a plane P or S wave. As the incident P wave (denoted as IP) encounters the fracture, its energy may be reflected as a P wave (RPP) or a converted S wave (RPS). When passing through the fracture, the incident P wave may be transmitted as a P wave (TPP) or a converted S wave (TPS), as shown in Fig. 14a. Similarly, the incident S wave (IS) may be reflected as an S wave (RSS) or a converted P wave (RSP), or transmitted as an S wave (TSS) or a converted P wave (TSP). We derive the energy densities of P and S waves to quantify wave energy as follows (Shapiro et al. 2000):

10 $$W_{{\text{P}}} = \left( {\frac{{\mu_{1} }}{2} + \mu_{2} } \right) \cdot \left( {{\text{div }}{\mathbf{u}}} \right)^{{2}} {\text{ and }}W_{{\text{S}}} = \frac{{\mu_{2} }}{2} \cdot \left( {{\text{curl }}{\mathbf{u}}} \right)^{2} ,$$

where *μ*_1_ and *μ*_2_ are the first and second Lamé constants, respectively, and **u** is the displacement at a receiver. The array of receivers is evenly distributed throughout the study domain with a separation distance of *λ*/300. Figure 14c gives an example of recorded signals of *W*_P_ (energy density for IP) and *W*_S_ (energy density for TPS). The recorded signals are transformed onto the frequency domain via fast Fourier transformation (FFT), from which the spectral amplitudes of waves are extracted at the central frequency. Thus, the reflection and transmission coefficients are derived as follows:

11 $$\left\{ \begin{gathered} R_{{{\text{P}} \to {\text{P}}}} = \sqrt {A_{{{\text{RPP}}}} /A_{{{\text{IP}}}} } \hfill \\ R_{{{\text{P}} \to {\text{S}}}} = \sqrt {A_{{{\text{RPS}}}} /A_{{{\text{IP}}}} } \hfill \\ T_{{{\text{P}} \to {\text{P}}}} = \sqrt {A_{{{\text{TPP}}}} /A_{{{\text{IP}}}} } \hfill \\ T_{{{\text{P}} \to {\text{S}}}} = \sqrt {A_{{{\text{TPS}}}} /A_{{{\text{IP}}}} } \hfill \\ \end{gathered} \right.{\text{ and }}\left\{ \begin{gathered} R_{{{\text{S}} \to {\text{P}}}} = \sqrt {A_{{{\text{RSP}}}} /A_{{{\text{IS}}}} } \hfill \\ R_{{{\text{S}} \to {\text{S}}}} = \sqrt {A_{{{\text{RSS}}}} /A_{{{\text{IS}}}} } \hfill \\ T_{{{\text{S}} \to {\text{P}}}} = \sqrt {A_{{{\text{TSP}}}} /A_{{{\text{IS}}}} } \hfill \\ T_{{{\text{S}} \to {\text{S}}}} = \sqrt {A_{{{\text{TSS}}}} /A_{{{\text{IS}}}} } \hfill \\ \end{gathered} \right.,$$

where *A* is the amplitude of recorded signals of energy density and the subscripts denote the corresponding types of waves. In this validation test, without loss of generality, we parameterize the model based on Table 1 and explore a series of dimensionless angular frequencies *ῶ* ranging from 0.01 to 1000. The corresponding fracture stiffness *κ* = *Zω*/*ῶ* varies from 0.3926 GP/m (for *ῶ* = 1000) to 39,256 GP/m (for *ῶ* = 0.01), given that the wavelength *λ* = 0.6 m. The incidence angle of incident P wave ranges from 10° to 60° with an interval of 10°. Since the incidence angle of S wave must be smaller than the critical angle (Gu et al. 2016), i.e., *θ*_c_ = arcsin(*V*_S_/*V*_P_) = 34.7°, we investigate the incidence angle of S wave within the range from 5° to 30° with a 5° interval. Figures 15 and 16 show the numerical results and analytical solutions of the coefficients for both incident P and S waves as a function of *ῶ*, which show a good agreement and thus further justify the validity of our model for simulating oblique wave propagation scenarios.

## List of Symbols

*α*: Roughness exponent
*A*: Wave amplitude of recorded signals of energy density
$A\left( {x_{i} ,y_{i} } \right)$: Wave amplitude at a point $\left( {x_{i} ,y_{i} } \right)$ when the wave travels in a heterogeneous medium
$A^{0} \left( {x_{i} ,y_{i} } \right)$: Wave amplitude at a point $\left( {x_{i} ,y_{i} } \right)$ when the wave travels in a homogeneous medium
*C*(Δ): Second-order front–front correlation function
**C**^*^: Damping matrix of the absorbing layer
*δ*: Separation distance between two adjacent receivers
*δ**l*: Measurement scale for a fractal surface
Δ: Distance of two points on a self-affine surface
d*t*: Time step in the numerical simulation
$d\left(\widetilde{t},{y}_{i}\right)$: Position of a point (i.e., a receiver) on the front of first-arrival waves with *y*_*i*_ as its *y* coordinate at dimensionless time $\widetilde{t}$
$\overline{{d\left( {\tilde{t}} \right)}}$: Average distance of points on the front of first-arrival waves from source line at $\widetilde{t}$
*D*: Fractal dimension
*ε*: Coefficient in *ψ*_max_, taking a value of 0.01
*E*: Young’s modulus of intact rock
**F**: External force vector
*η*: Mass proportional damping coefficient of the absorbing layer
*κ*: Specific stiffness of a fracture
*κ*_n_, *κ*_s_: Normal and shear specific stiffnesses of a fracture, respectively
*k*: Wavenumber of the incident wave
**K**: Stiffness matrix of solids
**K**^*^: Stiffness matrix of the absorbing layer
*λ*: Wavelength of the incident wave
*l*_c_: Correlation length
*l*_ele_: Average element size
*l*_min_, *l*_max_: Minimum and maximum fracture lengths, respectively
*L*: Size of the study domain
*L*_FFAW_(*δl*): Length of the front of first-arrival waves with *δl* as the measurement scale
*μ*_1_, *μ*_2_: First and second Lamé constants, respectively
**M**: Mass matrix of solids
*N*_*y*_: Number of receivers
*ν*: Poisson’s ratio of intact rock
*ξ*: Exponent for *ῶ* = 10^*ξ*^
*ξ*_c_: Critical exponent for *ῶ*_c_ = $10^{{\xi_{{\text{c}}} }}$
*θ*, *θ*_c_: Incidence angle for P/S waves and critical angle for incident S wave, respectively
*ρ*: Density of intact rock
*r*^2^: Coefficient of determination for a fitting curve
$R_{{{\text{P}} \to {\text{P}}}}$, $R_{{{\text{P}} \to {\text{S}}}}$: Reflection coefficients from an incident P wave to reflected P and converted S waves, respectively
$T_{{{\text{P}} \to {\text{P}}}}$, $T_{{{\text{P}} \to {\text{S}}}}$: Transmission coefficients from an incident P wave to transmitted P and converted S waves, respectively
$R_{{{\text{S}} \to {\text{S}}}}$, $R_{{{\text{S}} \to {\text{P}}}}$: Reflection coefficients from an incident S wave to reflected S and converted P waves, respectively
$T_{{{\text{S}} \to {\text{S}}}}$, $T_{{{\text{S}} \to {\text{P}}}}$: Transmission coefficients from an incident S wave to transmitted S and converted P waves, respectively
*σ*_n_, *σ*_s_: Normal and shear stresses of the fracture, respectively
$\sigma_{{\text{n}}}^{ + }$, $\sigma_{{\text{n}}}^{ - }$: Normal stress on either side of the fracture
$\sigma_{{\text{s}}}^{ + }$, $\sigma_{{\text{s}}}^{ - }$: Shear stress on either side of the fracture
*ς*: Stiffness reduction coefficient of the absorbing layer
*t*: Time
*t*^0^: Traveling time for wave arrival at a point in a homogeneous medium
*t*_*i*_: Traveling time for wave arrival at a receiver $\left( {x_{i} ,y_{i} } \right)$ in a heterogeneous medium
*t*_0_: Traveling time of wave arrival at the right boundary in intact rock
$$\widetilde{t}$$: Dimensionless time
${\widetilde{t}}_{\mathrm{b}}$: Dimensionless breakthrough time
**u**: Displacement
$u_{{\text{n}}}^{ + }$, $u_{{\text{n}}}^{ - }$: Normal displacement on either side of the fracture
$u_{{\text{s}}}^{ + }$, $u_{{\text{s}}}^{ - }$: Shear displacement on either side of the fracture
$U\left( {t_{i} ,x_{i} ,y_{i} } \right)$: Displacement of a receiver $\left( {x_{i} ,y_{i} } \right)$ at time *t*_*i*_ in a homogeneous medium
$U^{0} \left( {t^{0} ,x_{i} ,y_{i} } \right)$: Displacement of a receiver $\left( {x_{i} ,y_{i} } \right)$ at time *t*^0^ in a heterogeneous medium
*V*_P_, *V*_S_: Velocities of P and S waves, respectively
*χ*: Distance away from the boundary of the study area
*x*: Distance traveled by the wave
*y*_*i*_: Coordinate along the *y* axis
*ψ*: Attenuation factor of the absorbing layer
*ψ*_max_: Maximum attenuation factor of the absorbing layer
*ω*: Angular frequency of the incident wave
*ῶ*: Dimensionless angular frequency
*ῶ*_c_: Critical dimensionless angular frequency
*W*_P_, *W*_S_: Energy densities for P and S waves, respectively
*Z*: Seismic impedance of intact rock
